# Thermal and Mechanical Behavior of Wood Plastic Composites by Addition of Graphene Nanoplatelets

**DOI:** 10.3390/polym11081365

**Published:** 2019-08-19

**Authors:** Xingli Zhang, Jinglan Zhang, Ruihong Wang

**Affiliations:** 1 College of Mechanical and Electrical Engineering, Northeast Forestry University, Harbin 150040, China; 2 Key Laboratory of Functional Inorganic Material Chemistry, Heilongjiang University, Harbin 150010, China

**Keywords:** wood plastic composite, graphene nano-platelets, thermal property, mechanical property

## Abstract

Wood plastic composites (WPCs) incorporating graphene nano-platelets (GNPs) were fabricated using hot-pressed technology to enhance thermal and mechanical behavior. The influences of thermal filler content and temperature on the thermal performance of the modified WPCs were investigated. The results showed that the thermal conductivity of the composites increased significantly with the increase of GNPs fillers, but decreased with the increase of temperature. Moreover, thermogravimetric analysis demonstrated that coupling GNPs resulted in better thermal stability of the WPCs. The limiting oxygen index test also showed that addition of GNPs caused good fire retardancy in WPCs. Incorporation of GNPs also led to an improvement in mechanical properties as compared to neat WPCs. Through a series of mechanical performance tests, it could be concluded that the flexural and tensile moduli of WPCs were improved with the increase of the content of fillers.

## 1. Introduction

Wood plastic composites (WPCs) are replacement products of wood made by dispersing wood flour into polymer matrix, and are widely utilized in construction, packaging, and furniture products [1]. WPC products show an excellent thermal insulation property, which may also limit their application, such as in heated floors. A possible solution to improve the thermal properties of WPCs is the introduction of additives. Nanocarbon materials are the main candidates for this purpose owing to their superior thermal conductivity [2,3,4]. Among the nanocarbon materials, graphene nanoplatelet (GNPs) fillers have attracted intensive research interests to obtain a dramatic improvement in the thermal properties of polymer composites. Compared with other nanocarbon materials, the high specific surface area of GNPs helps a great deal to result in an effective dispersion degree of the fillers. In addition, GNPs as a thermally conductive filler could maintain or even improve the mechanical properties of the composites because of their own excellent mechanical strength [5,6,7].

Recently, extensive research on enhancing the heat transfer of polymers by adding GNPs has been reported. The thermal properties of epoxy/GNPs composites were studied through thermal conductivity measurements, and the results showed that the introduction of 20 wt % chemically-functionalized GNPs enhanced thermal conductivity 29 times relative to that of pure epoxy [8]. The modeling results of Khan et al. showed that the significantly high improvement of the cross-plane thermal conductivity of GNP-based polymer is ascribed to good thermal transmission through GNP networks in the polymer matrix [9]. Zhou et al. reported that the high aspect ratio of GNPs is one of the key factors contributing to higher thermal conductivity of epoxy with relatively lower filler contents [10]. The above experimental studies mainly focused on the application of GNPs in polymers, but the attention given to the introduction of GNPs into WPCs is still scarce.

Herein, a competitive WPC reinforced with GNPs is reported. The resulting WPCs exhibited the highest thermal conductivity compared to the values of neat WPCs. The influences of filler content and temperature on the thermal and mechanical properties of composites were also investigated in detail. 

## 2. Materials and Experiment

### 2.1. Materials Collection

Poplar wood fiber (WF) was obtained from Baiquan, Heilongjiang Province in China. Polyethylene (PE) (Fushun Petroleum Company, Fushun, China) was used as polyolefin matrix. Maleic anhydride grafted polyethylene (MAPE) (Rizhisheng New Materials Technology, Shanghai, China) was used as a compatibility agent to help the polymers bond together, and the weight content of MAPE in the total composite dry mass was never below 3%. The graphene nanoplatelets (GNPs) were purchased from Suzhou Rich Carbon Graphite Technology Company, with purity > 95%, thickness 1.0–1.77 nm, lamellar diameter 10–50 μm, surface area 360–450 m^2^/g.

### 2.2. Sample Preparation

WF was dried by a convection oven at 103 °C for 24 h to obtain 3% moisture content. The dried WF, PE, MAPE and GNPs were then blended in a high-speed mixer for 10 min. The weight contents of raw materials are listed in Table 1 (control means the original wood plastic composites and WPC3 means the composites adding 3 wt % GNPs). A co-rotating twin screw extruder (Xawax Science Technology Company, Nanjing, China) was used in extruding the blends. The temperature of the extruder barrel was set between 140 and 180 °C with the screw rotating speed set at 30 rpm. The extruded melts were manufactured using a hot-press machine (BY114×8, Jianhu Machinery Factory, Nanjing, China) for 2 min. Finally, the WPC sheets were molded into test specimens sized 100 mm (L) × 100 mm (W) × 4 mm (H). 

### 2.3. Sample Characterization

#### 2.3.1. Morphology Analysis

The microstructure of the GNPs was observed using a transmission electron microscope (TEM, 912 AB, Oberckochen, Munster, Germany) at an accelerating voltage of 100 kV. After sputtered with a thin layer of gold, the surface morphology of samples were taken using a scanning electron microscope (SEM, Quanta 200, FEI Company, Eindhoven, Netherlands) at an acceleration voltage of 12.5 kV.

#### 2.3.2. Thermal Properties

The heat transfer performance of the modified WPCs sample was determined using a TC-3100 thermal measuring instrument (Xiatech Group, Xi’an, China) base using a transient hot wire method. The specimen dimensions were 40 mm × 40 mm × 4 mm, and the test temperatures were 20–80 °C. The accuracy of thermal conductivity measurements was about ±3%. Thermogravimetric analysis of the WPCs was measured by Perkin Elmer Pyris 6 TGA for analyzing the thermal decomposition temperature of composites. The heating condition was in the temperature range of 50–700 °C, and heating rate was 10 °C/min. 

The limiting oxygen index (LOI) was measured according to ASTM D2863, and the apparatus used was a JF-3 oxygen index meter (Jiangning Analysis Instrument Company, Nanjing, China). The specimen dimensions used for the test were 130 × 10 × 3 mm^3^.

#### 2.3.3. Mechanical Properties

Flexural and tensile properties of samples were measured according to ASTM D638-2004 standard, using a universal mechanical machine (Regear Instrument Cooperation, Shenzhen, China). Unnotched impact strength was measured according to ASTM D4812-2004 standard, using a JJ-20 impact tester (Intelligent Instrument Cooperation, Changchun, China) at a speed of 5 mm/min. For each mechanical analysis, six samples were tested.

Dynamic mechanical analysis (DMA) was performed in a dual cantilever mode using a dynamic mechanical analyzer (TA Instruments Inc, New Castle, DE, USA). The heating condition was in the temperature range of −10 to 120 °C, and heating rate was 3 °C/min. 

## 3. Results and Discussion 

### 3.1. Morphological Properties

**Figure 1:**
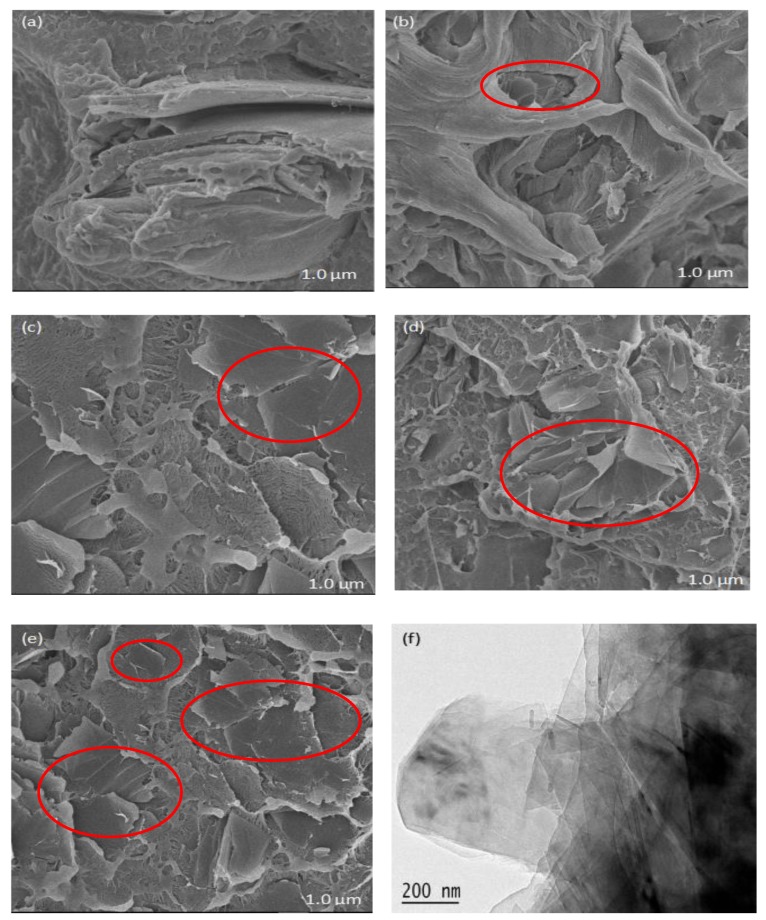
SEM and TEM micrographs of WPCs adding GNPs. (**a**) Control; (**b**) WPC3; (**c**) WPC6; (**d**) WPC9; (**e**) WPC12; (**f**) TEM image of GNPs.

This section shows the microstructure and the low-temperature fracture morphology of WPCs with an increase in GNPs content. As shown in the TEM images (Figure 1f), the general shape of GNPs is a semi-transparent film with twists and turns. In most cases, GNPs are in an overlapping state of multiple layers. The SEM images show that a stable heat conduction network chain is formed in the WPCs with the increase in GNP content, but in further increasing the packing material, the GNPs tended to form a higher structure, which lead to agglomeration in the PE matrix (see Figure 1d,e). Since the diffusion of phonons caused effective thermal conductivity in the GNPs, a good dispersion of GNPs in the PE matrix can steadily improve the thermal conductivity of the WPCs [11,12]. However, many GNPs overlap and agglomerates become defects in WPCs, which may disrupt the formation of network cross-linking and lead to a decrease in mechanical properties [13].

### 3.2. Thermal Properties

#### 3.2.1. Effect of GNPs Content on the Thermal Conductivity

**Figure 2:**
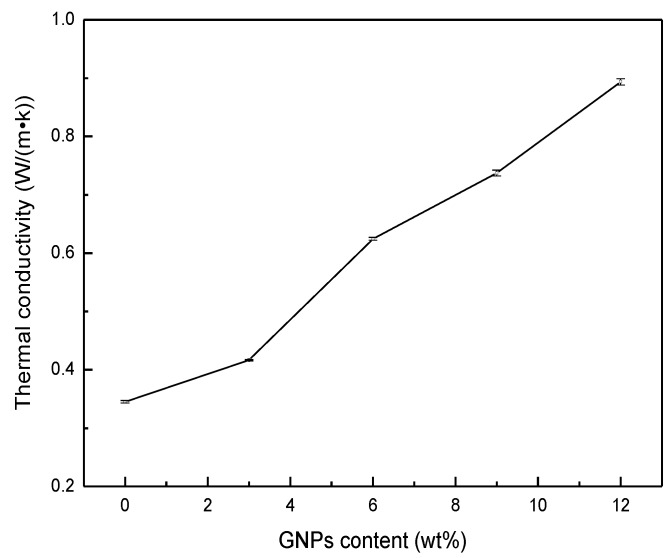
Thermal conductivity of the WPCs as a function of GNPs contents.

The WPCs samples with added GNPs exhibited a dramatic enhancement in thermal conductivity compared to neat WPCs, as shown in Figure 2. When the content of GNPs increased to 12 wt %, the thermal conductivity of WPCs increased by a factor of 258.9%. Compared to graphite [14], the addition of GNPs had a more significant enhancement effect on the heat transfer property of WPCs. Since the effective heat conduction in GNPs is attributed to phonon diffusion, the main method of modifying WPC heat conduction is also phonon heat conduction [15]. It can be found from SEM micrograph (Figure 1) that there are multiple interfaces between the GNPs fillers, WF, and PE matrix, which cause greater contact resistance and phonon scattering at interfaces. Due to the increase in the mass ratio of GNPs, the interfacial thermal contact resistance decreases, which minimizes the scattering of interfacial phonons and improves the thermal conductivity of the composites. Thus, better dispersion could lead to a decrease in the thermal contact resistance between fillers and polymer matrix, which are the key factors to exhibit higher thermal conductivity enhancement of nanocomposites. 

#### 3.2.2. Effect of Temperature on the Thermal Conductivity

**Figure 3:**
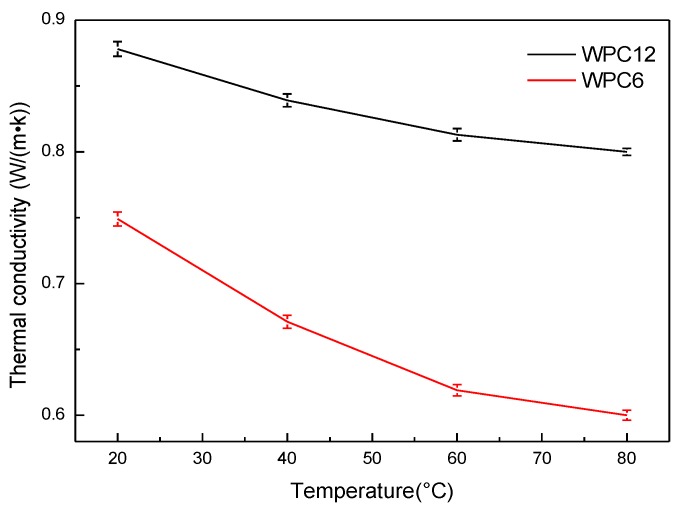
Thermal conductivity of the WPCs as a function of temperature.

Figure 3 shows the thermal conductivity behavior of WPCs as a function of temperature. The thermal conductivities of WPC6 and WPC12 decreased gradually when the temperature increased from 20 °C to 80 °C. The thermal conductivity of GNPs and PE have been experimentally or theoretically assessed [16,17,18]. Many results show that temperature adversely affects the diffusion of phonons, so the thermal conductivity of WPCs produce a declining trend due to the predominant decrease in the thermal conductivity of GNPs and PE with temperature. In addition, the data show that when the GNPs content is higher, the thermal conductivity is less affected by the temperature change, indicating that a high content of GNPs is helpful to form a stable thermal conduction network in the polymer matrix. Therefore, GNP filler may effectively reduce the influence of temperature change on the thermal conductivity of a composite. 

#### 3.2.3. Thermogravimetric Analysis (TG)

**Figure 4:**
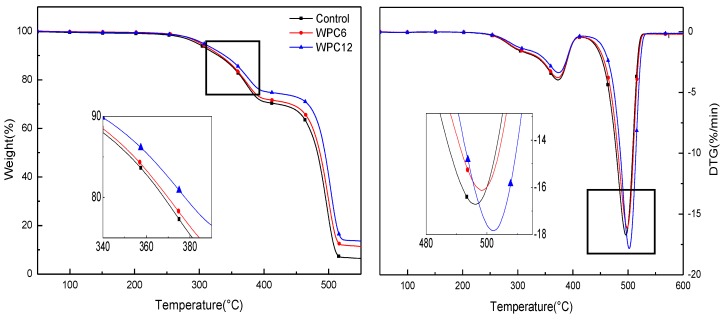
Mass loss curves and DTG curves of WPCs with added GNPs.

TGA/DTG profiles for test samples as a function of temperature are shown in Figure 4. TGA curves show that all samples exhibited similar thermal behavior. There are two times of weightlessness in the degradation process of samples. The first weight loss takes place at around 300–350 °C, which is attributed to the evaporation of wood components, such as hemicellulose, cellulose, and lignin. The second weight loss takes place at around 450–500 °C, which is caused by the degradation of the polymer matrix. Moreover, with the increase in GNPs content, the thermal degradation temperature of the composite gradually increased. The DTG curve also exhibits two derivative peaks, which are also related to the decomposition of wood fiber and PE, and the highest degradation temperature of the second peak is much higher than the first peak. Table 2 shows that the temperatures of 5%, 10% and 50% weight loss (T_5_, T_10_, T_50_) increase with increasing GNPs content. It is believed that the presence of GNPs is beneficial to the enhancement of thermal stability of the composites. 

#### 3.2.4. The Limiting Oxygen Index (LOI) Test

**Figure 5:**
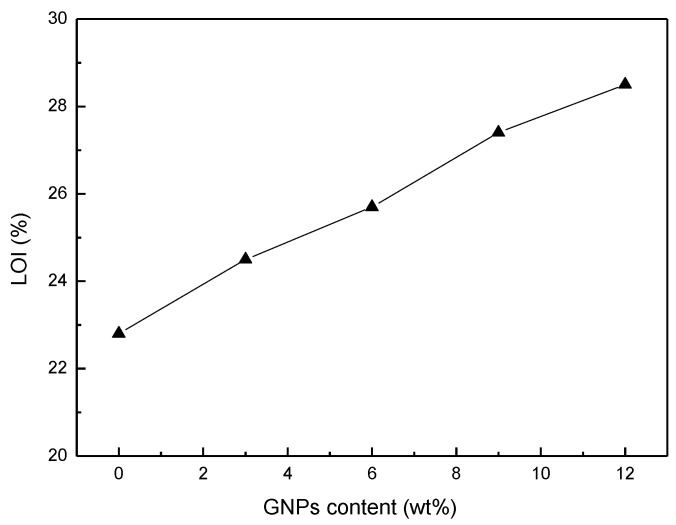
The LOI values of the WPCs with added GNPs.

As shown in Figure 5, the LOI values of WPCs with added GNPs are higher than those of neat WPCs, and increase with the GNP content. Previous reports demonstrated that graphene or its derivatives, as fire retardant nanofillers, could reduce the fire hazard of various polymers, as a protective intumescent carbonaceous char was formed on the surface of the materials [19,20]. Here the LOI test results confirm the conclusion that the addition of GNPs causes good fire retardancy in WPCs.

#### 3.2.5. Mechanical Properties

The mechanical properties of WPCs are significantly affected by GNPs content. As shown in Table 3, with increasing content of GNPs from 0 to 12 wt %, the flexural strength, flexural modulus and tensile modulus increase by 35.3%, 47.9% and 15.9%, respectively, but the tensile strength and impact strength of WPCs decrease slightly. It is well known that the adhesion and aggregation of nano-filler lead to defects in composites, as shown by SEM analysis (Figure 1), which is a major cause of instability in mechanics performance [21,22]. However, GNPs as filler have a beneficial impact on the flexural and tensile moduli of WPCs compared to other carbon nanomaterials. This may be explained by the excellent intrinsic mechanical resistance of GNPs. For flexural strength especially, the values decrease to an extent and then begin to increase gradually. The very large contacting interface between GNPs and PE matrix firstly cause a decrease of flexural strength. With increasing GNPs, their intrinsic mechanical properties play a leading role in improving the strength of WPCs.

**Figure 6:**
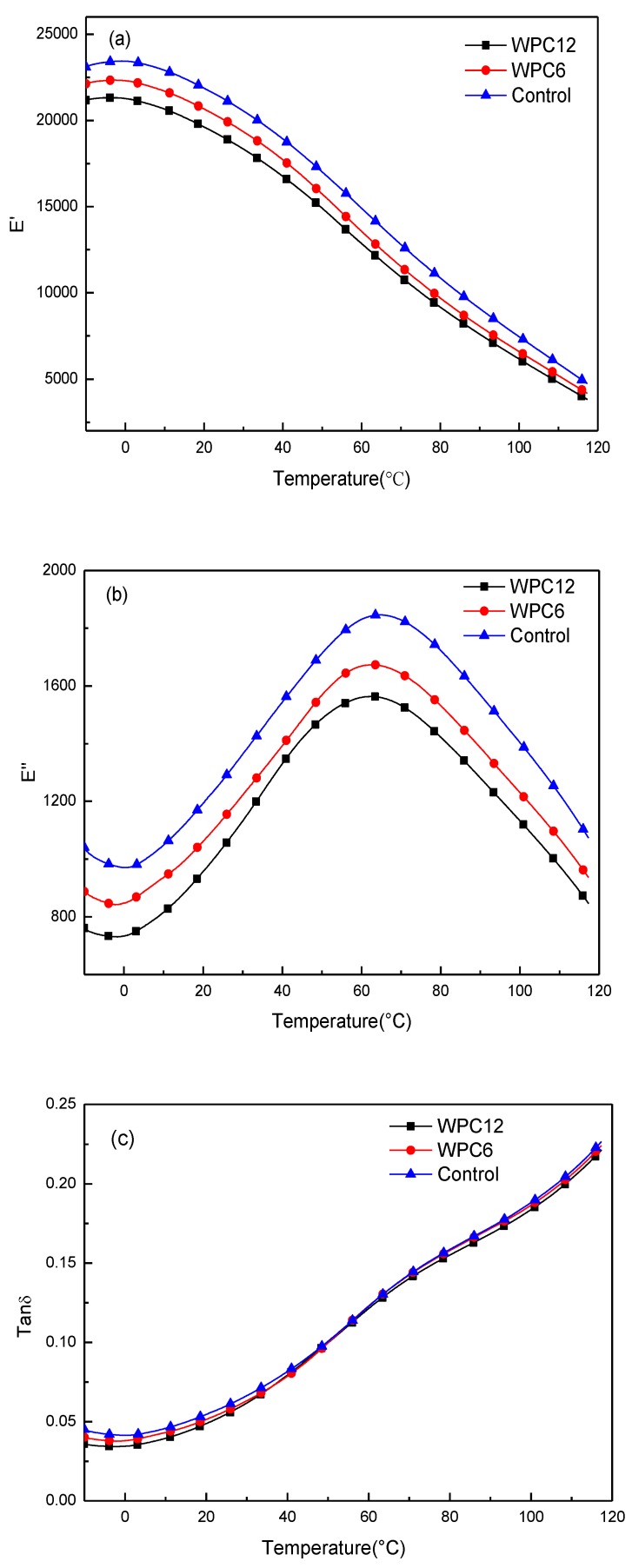
DMA curves of WPCs adding GNPs. (**a**) E′, (**b**) E″ and (**c**) tan δ.

The storage modulus (E′), loss modulus (E″), and tan δ determined using DMA are shown in Figure 6. The storage modulus (E′) tends to increase at first, then when the temperature exceeds 15 °C they exhibit a significant decreasing tendency. This result can be ascribed to the increase in molecular mobility of the PE chains at high temperatures [23]. In addition, the values of E′ also decrease with the increase in GNPs content, indicating that the presence of GNPs in the composite will increase the hindrance of the segmental motion of the polymer chains [24]. The loss modulus (E″) shows an opposite tendency compared to the storage modulus (in Figure 6b). The increase of the loss modulus may be due to the effects of interfacial interactions and entanglements [25]. The loss factor tan δ can be defined as the ratio of the loss modulus to the storage modulus, which is associated with the structural transformation in the composite [26]. In our study, three tan δ curves almost overlapped with the temperature increase, but the values were slightly different at the lower temperatures and tan δ decreased with an increase in GNP mass ratio. This phenomenon can be explained by the weak interfacial adhesion of GNPs in the PE matrix; however, the powerful mechanical properties of GNPs offset the shortage of interface between the polymer matrix and fillers, which is consistent with the mechanical test results.

## 4. Conclusions 

In summary, the thermal transport and mechanical properties of WPCs reinforced by an amount of GNPs are investigated. SEM observation shows that GNPs with higher content present a multilayer overlapping state or agglomerations in the polymer matrix. Therefore, homogeneous dispersion of fillers within the matrix should be the key factor in maximizing the filler’s effect. The thermal conductivity of the composite increases markedly with an increase in GNPs content, but decreases with increasing temperature. Additionally, TG analyses illustrate that WPCs modified by GNP fillers have better thermal stability than neat WPCs. The GNP fillers also have a positive effect on the mechanical properties of WPCs. The flexural strength of the composite decreases markedly at small amounts of GNPs, but begin to increase with more filler content. From the above study, it is evident that WPCs with added GNPs exhibited better improvement upon thermal conductivity, and avoid much of the decline in mechanical properties. This research also greatly extends the comprehensive utilization of WPCs.

## Figures and Tables

**Table 1:** Mass fraction of raw materials for wood-plastic composites.

Sample	WF (wt %)	PE (wt %)	MAPE (wt %)	GNPs (wt %)
Control	40 ± 1.5	57 ± 1.5	3 ± 0.1	0
WPC3	40 ± 2.0	54 ± 1.5	3 ± 0.2	3
WPC6	40 ± 1.4	51 ± 1.2	3 ± 0.1	6
WPC9	40 ± 1.7	48 ± 1.2	3 ± 0.1	9
WPC12	40 ± 2.0	45 ± 1.0	3 ± 0.2	12

**Table 2:** Thermal decomposition parameters for WPCs with added GNPs.

Sample	T_5_ (°C)	T_10_ (°C)	T_50_ (°C)	T_max_ (°C)
Control	296.85	329.00	482.53	373.53/496.33
WPC6	299.32	332.35	486.44	373.79/498.18
WPC12	299.56	339.12	494.07	374.14/502.14

**Table 3:** Mechanical properties WPCs adding GNPs.

WPC Type	Flexural Strength (MPa)	Flexural Modulus (GPa)	Tensile Strength (MPa)	Tensile Modulus (GPa)	Impact Strength (kJ/m^2^)
Control	45.89 ± 0.62	1.96 ± 0.02	22.52 ± 0.41	1.44 ± 0.03	9.71 ± 0.56
WPC3	41.59 ± 0.65	2.32 ± 0.02	21.05 ± 0.47	1.45 ± 0.04	8.75 ± 0.43
WPC6	42.43 ± 0.71	2.50 ± 0.03	20.53 ± 0.42	1.56 ± 0.06	8.42 ± 0.46
WPC9	46.51 ± 0.57	2.89 ± 0.05	20.12 ± 0.32	1.65 ± 0.05	8.40 ± 0.51
WPC12	47.51 ± 0.35	2.90 ± 0.04	20.10 ± 0.41	1.67 ± 0.04	8.32 ± 0.23

## References

[1] Wang Q.W., Wang W.H., Song Y.M. (2007). Wood-Plastic Composites and Products.

[2] Fang H.M., Bai S.L., Wong P. (2018). Microstructure engineering of graphene towards highly thermal conductive composites. Compos. Part A.

[3] Verdejo R., Bernal M.M., Romasanta L.J. (2011). Graphene filled polymer nanocomposites. J. Mater. Chem..

[4] Birm J.K., Fei Y., Han G., Wang Q.W., Wu Q.L. (2013). Mechanical and physical properties of core–shell structured wood plastic composites: Effect of shells with hybrid mineral and wood fillers. Compos. Part B.

[5] Teng C.C., Ma C.C. (2011). Thermal conductivity and structure of non-covalent functionalized graphene/epoxy composites. Carbon.

[6] Wejrzanowski T., Grybczuk M., Chmielewski M. (2016). Thermal conductivity of metal-graphene composites. Mater. Des..

[7] Huang C., Qian X., Yang R. (2018). Thermal conductivity of polymers and polymer nanocomposites. Mater. Sci. Eng. R.

[8] Chu K., Wang X., Li Y. (2018). Thermal properties of graphene/metal composites with aligned graphene. Mater. Des..

[9] Khan M.F.S., Alexander A.B. (2012). Graphene-multilayer graphene nanocomposites as highly efficient thermal interface materials. Nano Lett..

[10] Zhou T., Wang X., Cheng P., Wang T., Xiong D., Wang X. (2013). Improving the thermal conductivity of epoxy resin by the addition of a mixture of graphite nanoplatelets and silicon carbide microparticles. Express Polym. Lett..

[11] Xiang J., Drzal L.T. (2011). Thermal conductivity of exfoliated graphite nanoplatelet paper. Carbon.

[12] Shahil K.M.F., Balandin A.A. (2012). Thermal properties of graphene and multilayer graphene: Applications in thermal interface materials. Solid State Commun..

[13] Tang B., Hu G., Gao H. (2015). Application of graphene as filler to improve thermal transport property of epoxy resin for thermal interface materials. Int. J. Heat Mass Transf..

[14] Zhang X., Hao X., Hao J., Wang Q. (2018). Heat transfer and mechanical properties of wood-plastic composites filled with flake graphite. Therm. Acta.

[15] Malekpour H., Chang K.H., Chen J.C. (2014). Thermal conductivity of graphene laminate. Nano Lett..

[16] Gu J., Du J., Dang J., Geng W., Hu S., Zhang Q. (2014). Thermal conductivities, mechanical and thermal properties of graphite nanoplatelets/polyphenylene sulfide composites. RSC Adv..

[17] Sayanthan R., Wang X., Jay S., John W. (2017). Heat transfer performance enhancement of paraffin/expanded perlite phase change composites with graphene nano-platelets. Energy Procedia.

[18] Dilek K., Ismail H.T., Turhan C. (2003). Thermal conductivity of particle filled polyethylene composite materials. Compos. Sci. Technol..

[19] Guo C., Zhou L., Lv J. (2013). Effects of Expandable Graphite and Modified Ammonium Polyphosphate on the Flame-Retardant and Mechanical Properties of Wood Flour-Polypropylene Composites. Polym. Polym. Compos..

[20] Bin Y., Shi Y., Yuan B., Qiu S., Xing W., Hu W., Song L., Loc S., Hu Y. (2015). Enhanced thermal and flame retardant properties of flame-retardant-wrapped graphene/epoxy resin nanocomposites. J. Mater. Chem. A.

[21] Robertson D.H., Brenner D.W., Mintmire J.W. (1992). Energetics of nanoscale graphitic tubules. Phys. Rev. B.

[22] Brenner D.W., Shenderova O.A., Harrison J.A. (2002). A second-generation reactive empirical bond order (REBO) potential energy expression for hydrocarbons. J. Phys. Condens. Matter.

[23] Yavari F., Fard H.R., Pashayi K. (2011). Enhanced thermal conductivity in a nanostructured phase change composite due to low concentration graphene additives. J. Phys. Chem. C.

[24] Papageorgiou D.G., Kinloch I.A., Young R.J. (2017). Mechanical properties of graphene and graphene-based nanocomposites. Prog. Mater. Sci..

[25] Zhang X., Hao X., Hao J. (2018). Thermal and mechanical properties of wood-plastic composites filled with multiwalled carbon nanotubes. J. Appl. Polym. Sci..

[26] Rishi A.M., Kandlikar S.G., Gupta A. (2019). Improved wettability of graphene nanoplatelets (GNP)/copper porous coatings for dramatic improvements in pool boiling heat transfer. Int. J. Heat Mass Transf..

